# Up-regulation of pro-angiogenic pathways and induction of neovascularization by an acute retinal light damage

**DOI:** 10.1038/s41598-020-63449-y

**Published:** 2020-04-14

**Authors:** A. Tisi, G. Parete, V. Flati, R. Maccarone

**Affiliations:** Department of Biotechnology and Applied Clinical Sciences, University of L’Aquila, via Vetoio, Coppito 2, 67100 L’Aquila, Italy

**Keywords:** Retina, Retinal diseases

## Abstract

The light damage (LD) model was mainly used to study some of the main aspects of age related macular degeneration (AMD), such as oxidative stress and photoreceptor death. Several protocols of light-induced retinal degeneration exist. Acute light damage is characterized by a brief exposure (24 hours) to high intensity light (1000 lux) and leads to focal degeneration of the retina which progresses over time. To date there are not experimental data that relate this model to neovascular events. Therefore, the purpose of this study was to characterize the retina after an acute light damage to assess whether the vascularization was affected. Functional, molecular and morphological investigations were carried out. The electroretinographic response was assessed at all recovery times (7, 60, 120 days after LD). Starting from 7 days after light damage there was a significant decrease in the functional response, which remained low up to 120 days of recovery. At 7 days after light exposure, neo-vessels invaded the photoreceptor layer and retinal neovascularization occurred. Remarkably, neoangiogenesis was associated to the up-regulation of VEGF, bFGF and their respective receptors (VEGFR2 and FGFR1) with the progression of degeneration. These important results indicate that a brief exposure to bright light induces the up-regulation of pro-angiogenic pathways with subsequent neovascularization.

## Introduction

The retina is a high metabolic tissue and requires high concentrations of oxygen. The inner retina is supplied by the retinal vasculature, while the outer retina by the choroid^1,2^. The retinal vasculature is characterized by three parallel vascular plexuses, deriving from the central artery, that reaches the eye through the optic nerve and progressively branches along the retina^3^. The superficial plexus is located above the Ganglion Cell Layer (GCL); the inner plexus is located in the Inner Plexiform Layer (IPL), while the deep plexus is located in the Outer Plexiform Layer (OPL)^4^. The choroid lies behind the retinal pigment epithelium (RPE) and provides oxygen and nutrients by diffusion to the outer retina^5^. Any unbalance of the electrolytes alter retinal homeostatis and lead to vision impairment. The stimulus for retinal neovascularization is mainly due to local hypoxia or retinal ischemia and this causes blood vessel generation as an attempt to re-oxygenate the ocular tissue in several retinal pathologies^6–8^. In pathological conditions, the new vessels can infiltrate many ocular tissues, including vitreous, cornea, iris, lens, retina, optic disc, as well as the sub-retinal space. These vessels are leaky and are subject to bleeding, allowing potentially harmful factors to infiltrate the surrounding tissues^9^. Neovascularization often involves loss of vision, in fact it is known that abnormal ocular angiogenesis can occur in a wide spectrum of eye disorders, including AMD (age-related macular degeneration). AMD is a neurodegenerative disease and the leading cause of blindness worldwide. It consists of a progressive degeneration of the photoreceptors that are located in the macular region, an area of the retina that is responsible for acuity and colour vision^10^. It is important to note that AMD occurs in most cases as “atrophic AMD”, which is characterized by the deposition of drusen and the slow degeneration of RPE and subsequently of the photoreceptors, leading to a condition of extended retinal atrophy named “geographic AMD”. Another form of AMD is the “wet/exudative AMD”, also called “neovascular AMD” (nAMD)^11^. It is characterized by choroidal neovascularization (CNV) resulting in retinal detachment, photoreceptor degeneration and vision loss^10^. Patients affected by nAMD may also develop retinal atrophy after a few years from the beginning of the pathology^11^. The vascular endothelial growth factor (VEGF), also known as vascular permeability factor, is an angiogenic factor which has aroused particular interest in recent years, for its involvement in the angiogenic processes in many tissues. It has widely been shown that the isoform A of VEGF (VEGFA) plays a key role in nAMD pathogenesis. Additional angiogenic factors include insulin like growth factor 1 (IGF-1), basic fibroblast growth factor (bFGF), tumour necrosis factor-a (TNF-α), angiopoietin-2 and matrix metalloproteinases (MMPs)^12^. Also microglia cells play important roles in angiogenesis and maintenance of vascular homeostasis in the retina^13^. Activated microglia increase oxidative stress damage *in vitro* and promote apoptosis in pericytes. Moreover, microglia promote angiogenesis, migration, and proliferation of endothelial cells by destroying the tight junction and affecting the integrity of the vasculature^14^. Thus, microglia activation contribute to Blood-Retinal Barrier (BRB) breakdown and to neovascularization^15^. VEGF, in turn, exerts a feedback activity on microglia, enhancing their activation and migration.

Light damage (LD) is a common animal model used to study retinal degeneration *in vivo*^16^. The exposure to bright and continuous intense light induces in the end photoreceptor death and vision loss mainly through a mechanism that involves oxidative stress^17,18^. It is well known that an acute stress induced by 24 hours of light exposure leads to the photoreceptor degeneration in a specific region of the superior retina, that is particularly susceptible to damage, and which is identified in the literature as “hotspot”^19^. Moreover, the damaged area expands in size over time^18,19^. Recruitment of macrophages and their invasion in the photoreceptor layer have also been highlighted in LD models^18–21^. The features of the focal and progressive retinal damage, observed in this model, closely follow those observed during the progression of age-related macular degeneration, in the atrophic form of the human pathology^18,19^. On this basis, our goal was to assess whether this light damage model could also mimics some features of the exudative form of AMD. We demonstrated for the first time that acute light damage leads to the modulation of the most relevant pathways involved in nAMD, in particular the VEGF pathway, which is a fundamental feature in nAMD. This was associated with neovascularization, retinal degeneration and impairment of retinal function. To further deepen and characterize the degeneration processes due to acute light damage, we investigated its effects on retinal vascularization, which have been never investigated until now. We demonstrated for the first time that acute light damage leads to the modulation of the most relevant pathways involved in nAMD, in particular the VEGF pathway. This was associated with neovascularization, retinal degeneration and impairment of retinal function.

## Results

### Acute light damage causes a drastic reduction of visual function, that does not impair over time

We performed flash electroretinogram (fERG) recordings and analysed a-wave, b- wave and oscillatory potentials (OPs). The a-wave gives information about photoreceptor activity and in fact is the first wave occurring after a light stimulus. The b-wave is a positive potential which depends both on light stimulus and retinal adaptation and it is the overall response of the retina after light stimuli^22^. The OPs contribute to the rising slope necessary for the formation of the b-wave and they derive from the circuits of the inner retina^23^.

**Figure 1:**
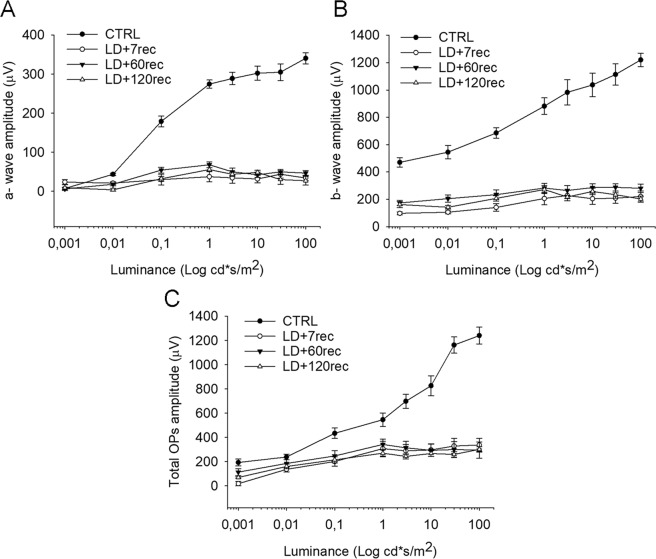
fERG recordings. (**A**) a-wave amplitude; (**B**) b-wave amplitude; (**C**) OPs total amplitude. No significant differences in the electrical response of the retina were observed after light exposure over time. Data are expressed as mean ± S.E. Statistical analysis was performed by one-way ANOVA test followed by Tukey test (n = 8). CTRL (Control); LD + 7rec (Light damage + 7 days of recovery); LD + 60rec (Light damage + 60 days of recovery); LD + 120rec (Light damage + 120 days of recovery).

In pathological conditions, especially when retinal degeneration occurs, the three parameters described above are affected and their amplitudes decrease^24,25^. Accordingly, we observed a remarkable deflection in a-wave (Fig. 1A), b-wave (Fig. 1B) and oscillatory potentials (Fig. 1C) when comparing the LD + 7rec, LD + 60rec and LD + 120rec groups to the Control group. No significant differences, instead, were observed between the three LD groups (Fig. 1). In fact, light damage provides an early acute stress that causes retinal degeneration, leading to impaired function^24,26–30^. The early injury factors are reduced over time after LD, but photoreceptors continue to die. In agreement with these findings, we did not observe changes in the electrical response between 7, 60 and 120 days of recovery.

### Acute light damage leads to VEGFA and VEGFR2 up-regulation over time

**Figure 2:**
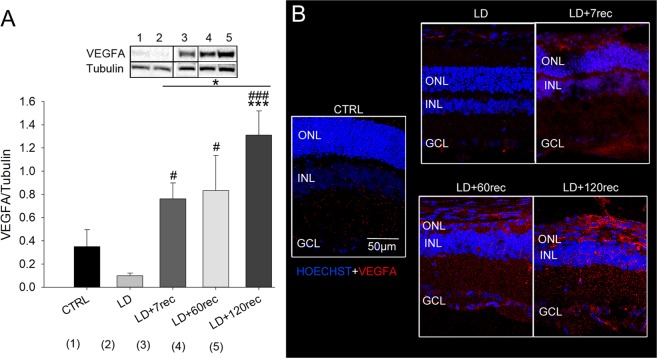
VEGFA analysis. (**A**) Western Blot analysis of VEGFA on eye cup samples of all the experimental groups. Statistical analysis was performed by one-way ANOVA test followed by Tukey test. Data are shown as mean ± SE (n = 4). *p < 0,05; **p < 0,01; ***p < 0,001 versus Control. ^#^p < 0,05; ^##^p < 0,01; ^###^p < 0,001 versus LD. Original western blot presented is available in Supplementary Fig. S1. (**B**) Confocal images of retinal cryosections immunolabelled for VEGFA (red). Nuclei were stained with Bisbenzimide (blue). The images refer to the central dorsal area of the retina. VEGFA increased in the rat retina after light exposure over time. CTRL (Control); LD (Light damage); LD + 7rec (Light damage + 7 days of recovery); LD + 60rec (Light damage + 60 days of recovery); LD + 120rec (Light damage + 120 days of recovery); ONL (Outer nuclear layer); INL (Inner nuclear layer); GCL (Ganglion cell layer).

The common feature of all the retinal diseases involving abnormal neovascularization, such as nAMD, is the up-regulation of VEGF. In fact many drugs for nAMD treatment, so far, have been developed with the aim of targeting VEGF^31^. Therefore, the first step to investigate vascular-related events was the analysis of the VEGF protein level. Western Blot analysis (Fig. 2A), showed that VEGF-A level was not altered soon after LD, if compared to the Control. Instead, it was upregulated over time in LD + 7rec (p = 0,010), LD + 60rec (p = 0,036) and LD + 120rec (p < 0,001) groups with respect to LD. The LD + 120 days group showed the most significant increase of VEGF, suggesting that the degeneration events occurring during the long-term recovery are involved in VEGF-A upregulation. In fact, in the LD + 120rec VEGF was significantly higher also compared to the control (p < 0,001) and the LD + 7rec group (p = 0,024). To support the western blot analysis, we also performed anti-VEGFA immunofluorescence staining on retinal cryosections together with bisbenzimide (Hoechst) staining (Fig. 2B). Through confocal image analysis we observed an up-regulation of VEGFA levels spreading within all the retinal layers. All the images were acquired by maintaining the same settings for the acquisition in order to avoid artefacts. The red signal deriving from anti-VEGFA immunostaining increased over time, consistently with the western blot analysis. Looking at the nuclei, 7 days after LD there was the occurrence of “rosettes” in the outer nuclear layer (ONL), that is the photoreceptor layer. This is a well known hallmark of retinal degeneration^32^. Over time the photoreceptor layer became more and more thinner as a consequence of the time elapsed after light exposure^18^. This data supports the concept that degeneration processes are ongoing although a long time after light exposure has passed.

**Figure 3:**
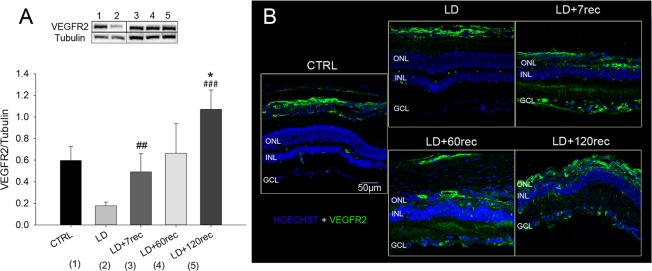
VEGFR2 analysis. (**A**) Western Blot analysis of VEGFR2 on eye cup samples of all the experimental groups. Statistical analysis was performed by one-way ANOVA test followed by Tukey test. Data are shown as mean ± SE (n = 4). *p < 0,05; **p < 0,01; ***p < 0,001 versus Control. ^#^p < 0,05; ^##^p < 0,01; ^###^p < 0,001 versus LD. Original western blot presented is available in Supplementary Fig. S2. (**B**) Confocal images of retinal cryosections immunolabelled for VEGFR2 (green). Nuclei were stained with Bisbenzimide (blue). The images refer to the central dorsal area of the retina. VEGFR2 increased in the rat retina after light exposure over time. CTRL (Control); LD (Light damage); LD + 7rec (Light damage + 7 days of recovery); LD + 60rec (Light damage + 60 days of recovery); LD + 120rec (Light damage + 120 days of recovery); ONL (Outer nuclear layer); INL (Inner nuclear layer); GCL (Ganglion cell layer).

In order to better characterize the role of the VEGFA signalling in the light damaged retina, we also quantified its receptor VEGFR2. The Western Blot analysis (Fig. 3A) showed that VEGFR2 was significantly overexpressed at 7 days of recovery (p = 0,002) compared to its levels observed soon after LD. Interestingly, it remained stable at 60 days after LD but it was further upregulated at 120 days compared to the control (p = 0,025) and the LD (p < 0,001) groups. The immunohistochemical analyses (Fig. 3B) showed that VEGFR2 in healthy adult albino retina was mainly present in the choroid, due to its ability to promote permeability of endothelial cells. A faint signal was also observed in the outer plexiform layer^33^. Our data clearly show that VEGFR2 was upregulated after LD and it also spread along the retinal layers. Taken together, these data indicate that both VEGF and VEGFR2 were up-regulated as consequence of light exposure. Their expression could depend on the degeneration processes ongoing after light exposure.

### Acute light damage leads to bFGF and FGFR1 up-regulation over time

bFGF is another important pro-angiogenic factor that acts by stimulating endothelial cell proliferation and migration through a crosstalk with VEGF. It is well known that an increase of bFGF expression induces VEGF up-regulation in endothelial cells forming capillaries^34^. On the other hand, it has been suggested that FGF-induced angiogenesis requires activation of the VEGF system^35^. Indeed, numerous studies have shown that bFGF signaling regulates the VEGF pathway at more than one level. In fact, bFGF induces the expression of VEGF in endothelial and stromal cells^36^.

**Figure 4:**
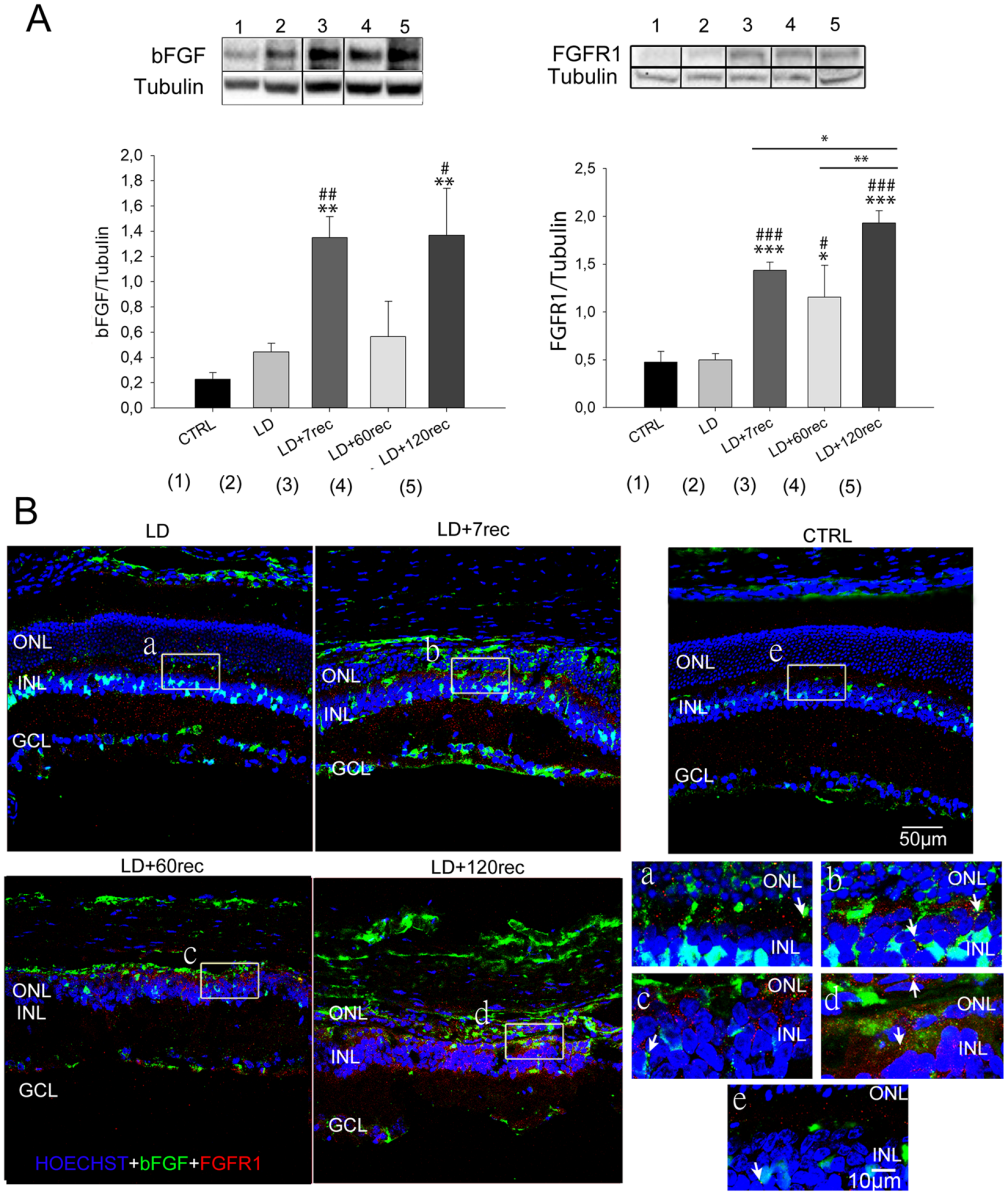
bFGF and FGFR1 analysis. (**A**) bFGF (left) and FGFR1 (right) analysis by Western Blot. Statistical analysis was performed by one-way ANOVA test followed by Tukey test. Data are shown as mean ± SE (n = 4). *p < 0,05; **p < 0,01; ***p < 0,001 versus Control. ^#^p < 0,05; ^##^p < 0,01; ^###^p < 0,001 versus LD. Original western blots presented are available in Supplementary Fig. S3. (**B**) Representative confocal images of retinal cryosections immunolabelled for bFGF (green) and FGFR1 (red). Nuclei were stained with Bisbenzimide (blue). (a–e) High magnification of a random area of the sections showing co-localization in yellow (white arrows) of bFGF and FGFR1. Both bFGF and FGFR1 increased in the rat retina after light damage over time. CTRL (Control); LD (Light damage); LD + 7rec (Light damage + 7 days of recovery); LD + 60rec (Light damage + 60 days of recovery); LD + 120rec (Light damage + 120 days of recovery); ONL (Outer nuclear layer); INL (Inner nuclear layer); GCL (Ganglion cell layer).

On this basis we analyzed the amount of bFGF and its receptor in the retinas of light damaged animals. The Western Blot analysis (Fig. 4A) showed that there was an overall upward trend both in bFGF and in FGFR1 expression due to the long term recovery after LD. In particular, bFGF significantly increased both in LD + 7rec and LD + 120rec compared to the control (LD + 7rec:p = 0,006; LD + 120rec:p = 0,008) and to the LD (LD + 7rec:p = 0,008; LD + 120rec:p = 0,014) groups. The FGFR1 analysis appeared to be even more interesting: we observed the same upward trend shown for bFGF. The three recovery groups showed a significant up-regulation of FGFR1 compared to the control (LD + 7rec:p < 0,001; LD + 60rec:p = 0,042; LD + 120rec:p < 0,001). A significant increase of FGFR1 expression was found in the LD + 7rec (p < 0,001), LD + 60rec (p = 0,026) and LD + 120rec (p < 0,001) groups when compared to the LD. Moreover, as for VEGFA and VEGFR2, the LD + 120rec group showed the highest up-regulation of FGFR1, which resulted to be significant increased also compared to the LD + 7rec (p = 0,023) and LD + 60rec (p = 0,009) groups. Subsequently, we analyzed the localization of bFGF and FGFR1 by immunofluorescence on retinal cryosections (Fig. 3B). Confocal image analysis of immunolabelled retinas were consistent with the western blot analysis, as an increasing signal deriving from bFGF and FGFR1 was observed over time. It is also shown a detail of each group where it is possible to appreciate the colocalization between bFGF and its receptor (Fig. 4B(a–e) – white arrows), indicating that the signalling pathway is likely activated.

### Acute light damage causes early neovascularization and long-term microglia activation

**Figure 5:**
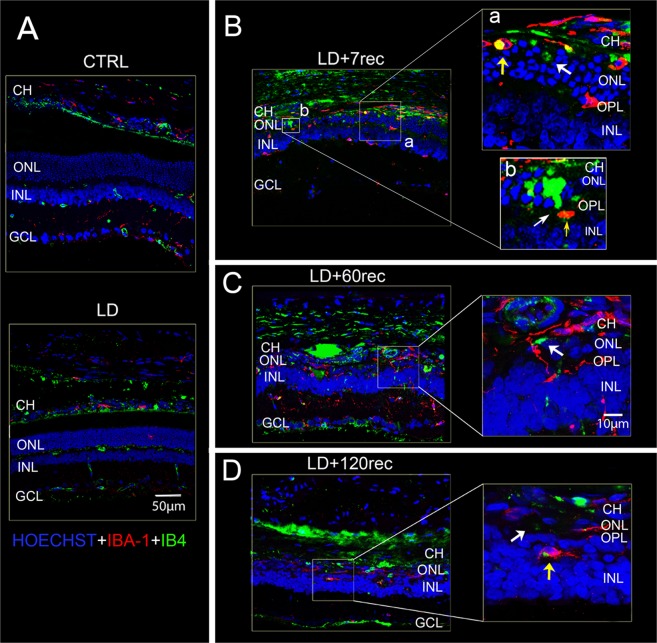
Isolectin B4 staining and anti-IBA-1 immunostaining. (**A**–**D**) Confocal images of retinal sections labelled for Isolectin IB4 (green) and IBA1 (red) showing vessels infiltration (white arrow) and activated microglia (yellow arrow) in the ONL; (**A**) CTRL and LD group: in which choroidal vessels infiltration and microglia activation were not observed; (**B**) LD + 7rec group; (a,b) zoom of LD + 7rec group; (**C**) LD + 60rec group; (**D**) LD + 120rec group. Nuclei were stained with Bisbenzimide. The images refer to the dorsal area of the retina. Neovascularization in the outer nuclear layer was highlighted starting 7 days after light exposure. Microglia were found to be activated up to 120 days after light damage, surrounding the new choroidal vessels infiltrating in the photoreceptor layer. CTRL (Control); LD (Light damage); LD + 7rec (Light damage + 7 days of recovery); LD + 60rec (Light damage + 60 days of recovery); LD + 120rec (Light damage + 120 days of recovery); CH (Choroid); ONL (Outer nuclear layer); OPL (outer plexiform layer); INL (Inner nuclear layer); GCL (Ganglion cell layer).

**Figure 6:**
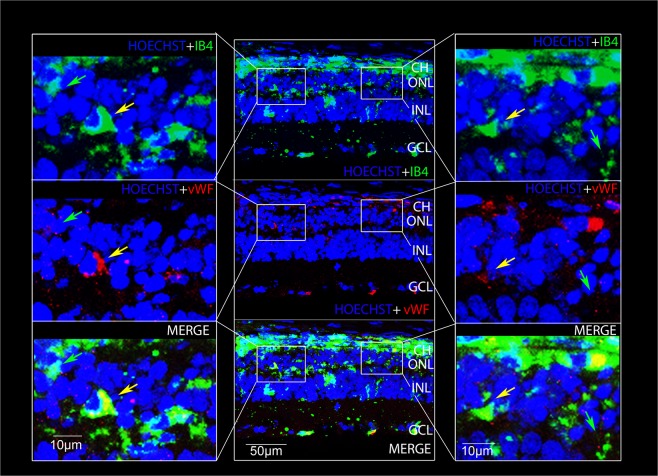
Isolectin B4 staining and anti-Von Willebrand immunostaining. Confocal images of retinal cryosections labelled for Isolectin IB4 (green) and anti-vWF (red) showing neoangiogenesis (yellow arrows) and activated microglia (green arrows) in the ONL after 7 days from LD. Nuclei were stained with Bisbenzimide. The images refer to the dorsal area of the retina. CH (Choroid); ONL (Outer nuclear layer); INL (Inner nuclear layer).

From the results described above it is possible to state that both VEGF and FGF are up-regulated and that they probably cooperate to promote angiogenesis. Activated microglia contribute to maintenance of vascular function and to angiogenesis in the retina^13^. In fact, it has been shown that activated microglia express VEGF^14^ and promote migration of endothelial cells and angiogenesis, contributing to neovascularization^15^. For these reasons microglial cells are often found surrounding the new vessels. On this basis, we proceeded with morphological analysis of the vasculature in order to evaluate the activation state of microglia cells and their alterations in the light damaged retina. We first focused on the presence of neovascularization in the ONL layer, since the absence of blood vessels in this layer is fundamental to allow normal vision, while their presence is a key feature involved in several retinal pathologies^37^. For this purpose, retinal cryosections of each experimental group were marked with Isolectin B4 (IB4). Although IB4 is largely employed to identify endothelial cells, it also detects activated microglia both in rodents and humans^38,39^. Therefore, in order to discriminate endothelial cells from activated microglia, we performed the isolectin staining together with anti-IBA-1 (Ionized calcium binding adaptor molecule 1, a selective marker for microglia) immunostaining (Fig. 5) and with anti-vWF (Von Willebrand Factor (Fig. 6), a well known specific endothelial marker^40^.

As shown in Fig. 5A, immediately after light exposure there were not activated microglia. Starting at 7 days after LD (Fig. 5B–D), instead, we observed the presence of activated microglia cells (yellow arrows) thanks to the co-localization of IBA1 (red) and Isolectin IB4 (green) resulting in a yellow signal. Here, we demonstrate for the first time that microglia are active after 120 days from light exposure (Fig. 5D). This data is in accordance with the progression of the retinal degeneration over time after light exposure. Moreover, by performing confocal microscopy analysis we were able to discriminate possible vessels (green) from resident microglia (red) and activated microglia in the photoreceptor layer (yellow) (Fig. 5B–D). After 7 days from light damage signs of neoangiogenesis were observed into the photoreceptor layer (Fig. 5B(a,b)). Over 7 days from light exposure, the degeneration processes continued and the photoreceptor layer got thinner, becoming almost degenerated up to 120 days after LD (Fig. 5C,D). Consequently, the choroid got closer to the retinal vasculature, present in the outer plexiform layer, that is the deep retinal vessels plexus, and remained adherent.

To prove more confidently the presence of vessels in the ONL, we also performed a double staining with IB4 and anti-Vwf (Fig. 6), which allows to exclude a microglial sub-population with IBA-1^−^/CD68^+^ phenotype^41^. The two signals clearly co-localized in the photoreceptor layer (yellow arrows) confirming the presence of blood vessels. Moreover, consistently with previous results, some IB4 signals did not co-localize with vWF, due to the presence of activated microglia (green arrows). To further confirm neoangiogenesis in the ONL layer, we also excluded the signal deriving from lipofuscin, or retinal debris. In fact, it is well known that during retinal degeneration lipofuscin accumulates in the subretinal region. It is characterized by an autofluorescent emission which covers a wide spectrum of wavelengths and which therefore could create confusion in the identification of new vessels^42^. On this basis, we discriminated them from retinal debris by anti-vWF immunostaining and acquiring the images by 488 and 594 excitation wavelengths, as shown in Supplementary Fig. S4.

### Acute light damage leads to vascular alterations in the retinal plexuses which are surrounded by activated microglia

Based on the observations on retinal cryosections, we moved on to study the vascular network present in the retinal plexuses. To investigate the retinal vasculature, we performed retinal whole mounts labelled with Isolectin B4. As shown in Supplementary Fig. S5, while performing whole mount analyses we spotted in green both amoeboid and ramified corpuscles (white arrows), showing a clear morphology reminiscent of microglia cells. As mentioned above, Isolectin B4 binds not only the endothelial cells but also to activated microglia^43^. For this reason, that signal was excluded from the whole mount analysis. Activated microglia was highlighted around the retinal vessels starting 7 days after light exposure, supporting the evidence that microglia could be involved in vascular alterations in the light damaged model.

**Figure 7:**
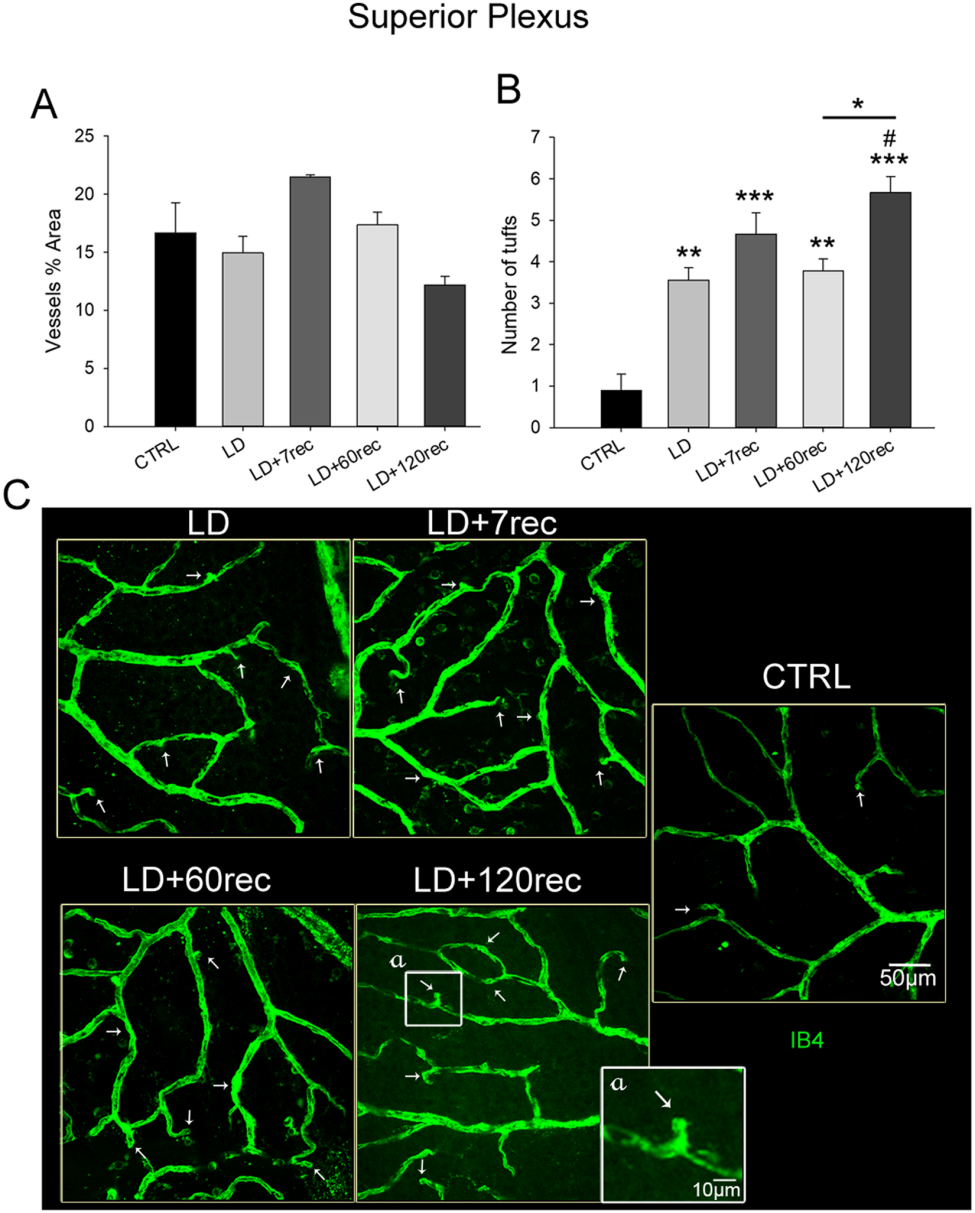
Analysis of the superior plexus vessels. (**A**) Vessels percentage area and (**B**) number of tufts of neovascularization. Histograms are shown as means ± S.E (n = 4); Statistical analysis was performed by one-way ANOVA test followed by Tukey test. *p < 0,05; **p < 0,01; ***p < 0,001 versus Control. ^#^p < 0,05; ^##^p < 0,01; ^###^p < 0,001 versus LD. (**C**) Representative confocal images of whole mounted retinas stained with Isolectin B4 showing the superior plexus of the central dorsal retina; (a) zoom on a tuft. The white arrows highlight the tufts of neovascularization. The number of tufts of neovascularization increased in the retinal superior plexus after light damage over time. CTRL (Control); LD (Light damage); LD + 7rec (Light damage + 7 days of recovery); LD + 60rec (Light damage + 60 days of recovery); LD + 120rec (Light damage + 120 days of recovery).

Starting from the superior plexus (corresponding to the ganglion cell layer) up to the deep retinal plexus (corresponding to the outer plexiform layer) the vessels percentage area and the number of tufts of neovascularization were analyzed. The analysis was focused on the central dorsal retina, that is the degenerating area after light damage. Tufts were counted considering the vessel bulges (Fig. 7C(a)), which are known to be a hallmark of neovascularization^38,44,45^.

In the superior plexus changes of the vessels percentage area were not found (Fig. 7A). The number of tufts of neovascularization, instead, resulted in a statistically significant increase over time (Fig. 7B). Immediately after LD, the number of tufts (Fig. 7B) was already significantly increased if compared to the control group (p = 0,004), although no differences in pro-angiogenic factors (and their receptors) were highlighted (Figs. 2, 3 and 4). The number of tufts was still significantly higher after 7 (p < 0,001), 60 (p = 0,003) and 120 (p < 0,001) days compared to the control. After 120 days from light exposure the number of tufts was even higher compared to LD (p = 0,02) and to the LD + 60rec (p = 0,038) groups. In Fig. 7C representative confocal images of the superior plexus of each experimental group are shown.

**Figure 8:**
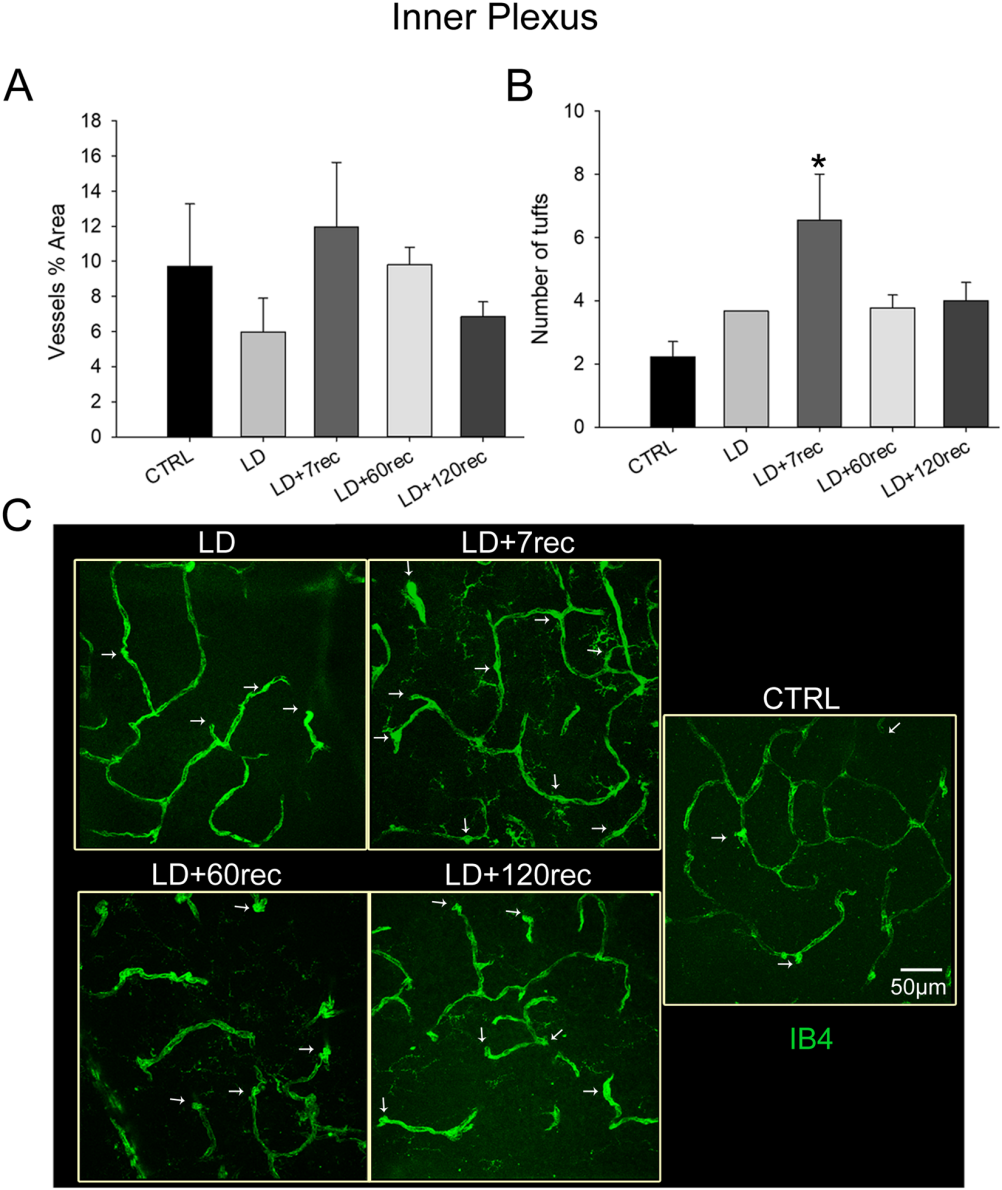
Analysis of the inner plexus vessels. (**A**) Vessels percentage area and (**B**) number of tufts of neovascularization. Histograms are shown as means ± S.E (n = 4); Statistical analysis was performed by one-way ANOVA test followed by Tukey test. *p < 0,05; **p < 0,01; ***p < 0,001 versus Control. ^#^p < 0,05; ^##^p < 0,01; ^###^p < 0,001 versus LD. (**C**) Representative confocal images of whole mounted retinas, stained with Isolectin B4, showing the inner plexus of the central dorsal retina. The white arrows highlight the tufts of neovascularization. The number of tufts of neovascularization increased in the retinal inner plexus after 7 days from light exposure and returned to normal conditions after a longer time of recovery. CTRL (Control); LD (Light damage); LD + 7rec (Light damage + 7 days of recovery); LD + 60rec (Light damage + 60 days of recovery); LD + 120rec (Light damage + 120 days of recovery).

In the inner plexus (Fig. 8), which corresponds to the inner plexiform layer and to the inner nuclear layer, no differences in the vessels percentage area were found (Fig. 8A), as well as in the superior plexus (Fig. 7A). The number of tufts (Fig. 8B) was significantly increased only in the LD + 7rec group compared to the control (p = 0,015).

**Figure 9:**
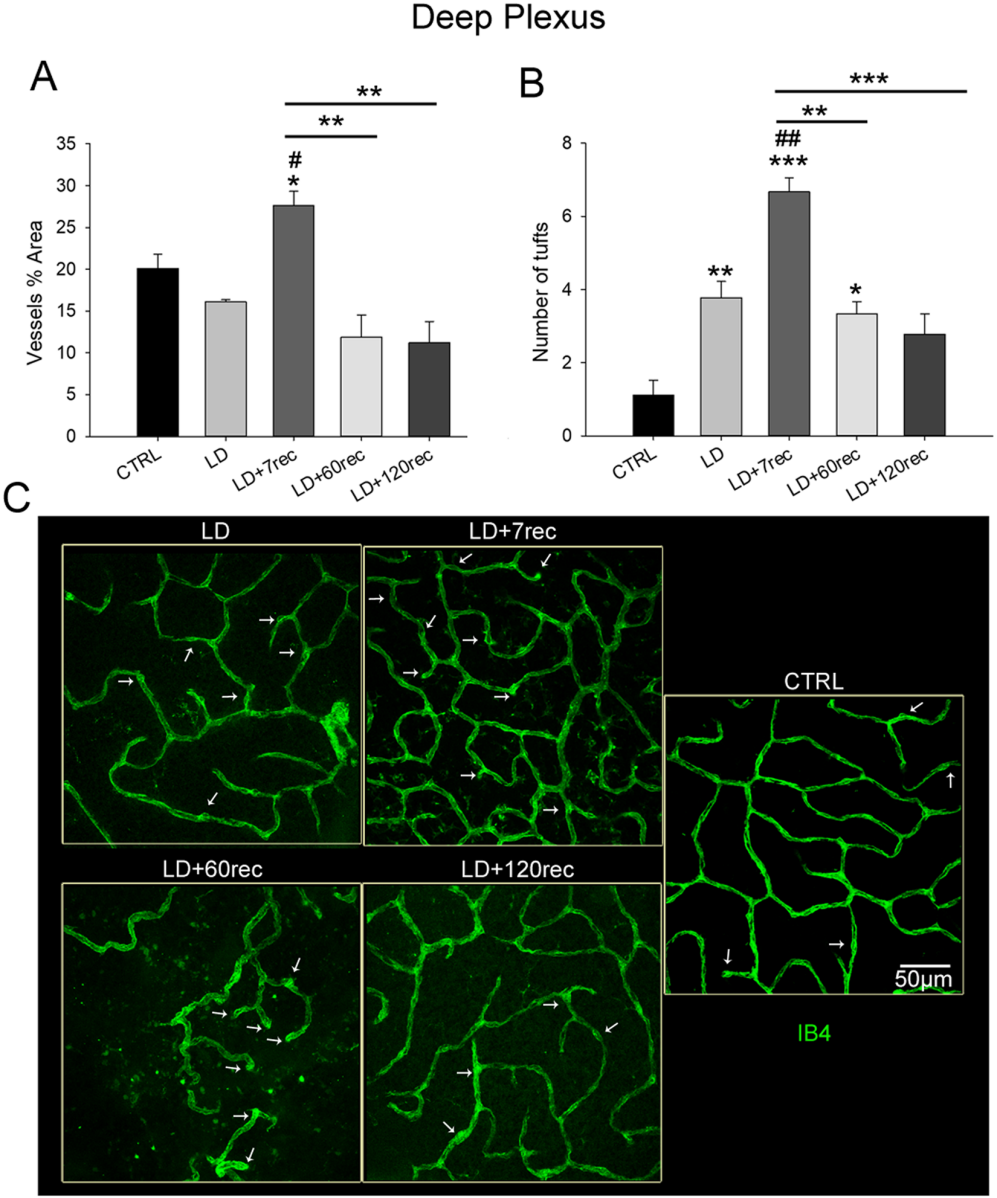
Analysis of the deep plexus vessels. (**A**) Vessels percentage area and (**B**) number of tufts of neovascularization. Histograms are shown as means ± S.E (n = 4); Statistical analysis was performed by one-way ANOVA test followed by Tukey test. *p < 0,05; **p < 0,01; ***p < 0,001 versus Control. ^#^p < 0,05; ^##^p < 0,01; ^###^p < 0,001 versus LD. (**C**) Representative confocal images of whole mounted retinas, stained with Isolectin B4, showing the deep plexus of the central dorsal retina. The white arrows highlight the tufts of neovascularization. The vessels percentage area was significantly increased in the deep plexus of LD + 7rec group compared to the control. The number of tufts of neovascularization increased in the retinal deep plexus up to 7 days from light damage and progressively decreased over time. CTRL (Control); LD (Light damage); LD + 7rec (Light damage + 7 days of recovery); LD + 60rec (Light damage + 60 days of recovery); LD + 120rec (Light damage + 120 days of recovery).

Interestingly, in the deep plexus, corresponding to the outer plexiform layer (OPL), we found that the vessels percentage area (Fig. 9A) was significantly increased 7 days after light damage if compared to the control (p = 0,036), the LD (p = 0,014), the LD + 60rec (p = 0,002) and LD + 120rec (p = 0,003) groups. This was clearly visible also by observing the vessels on whole mounted retinas (Fig. 9C). In fact, in the LD + 7rec group the vessels network appeared to be denser, consistently with the quantitative analysis of the vessels percentage area. The number of tufts (Fig. 9B), instead, was significantly increased in the deep plexus immediately after light exposure compared to the control. The tufts amount in the deep plexus was already significantly higher immediately after light exposure (p = 0,009) compared to the control and was the highest at 7 days after light damage compared to all the other experimental groups (p = 0,006 versus LD; p = 0,002 versus LD + 60rec; p < 0,001 versus LD + 120rec). After 60 days from light damage the number of tufts decreased compared to the LD + 7rec group, but was still significantly higher compared to the control (p = 0,029). After 120 days from the damage the number of tufts was not different from the control group. Interestingly, by looking at the vessels morphology (Fig. 9C), after 60 days from light damage the vessels network appeared disorganized and disrupted. Instead, after 120 days, a recovery in the vessels network in the deep plexus was observed.

## Discussion

Acute light damage (LD) is a model of retinal degeneration used worldwide to study some of the main features of AMD. To better characterize the acute LD model, in this study we investigated for the first time the vascular events underlying light-induced retinal degeneration. The findings of this study can relate the acute LD model to fundamental aspects of patients suffering from neovascular AMD: neurodegeneration and impairment of retinal function, VEGFA up-regulation, neovascularization in the photoreceptor layer and in the end retinal atrophy. However, further studies need to be performed in order to investigate the origin of the vessels invading the photoreceptor layer and verify whether choroidal neovascularization occurs as well as in human nAMD.

In particular, our data show that a brief light exposure (24 h) induces an acute stress in the retina of albino rats, leading to neovascularization and progressive retinal degeneration up to 4 months. Although further studies need to be performed, we can speculate that these new vessels may possibly connect with the vessels of the deep plexus thus leading to the formation of anastomosis, as previously demonstrated in another model of chronic light damage^46^. In fact, we demonstrated that the new vessels crossed the outer nuclear layer reaching the outer plexiform layer. Neovascularization occurred also within the vessel retinal plexuses as a consequence of light damage. Moreover, this study demonstrated for the first time that light damage induces VEGF (and other pro-angiogenic factors) up-regulation with subsequent neovascularization, as observed starting 7 days after light damage. The infiltration of new vessels in the photoreceptor layer, together with the increase of vascularization in the neuroretina, are certainly involved in the progression of the degeneration of the light damage model. In fact, in our model the late stages after light damage lead to an extended retinal atrophy, although no differences in the retinal function were highlighted. The photoreceptor layer became increasingly thin and degeneration lesions, named “rosettes”, occurred in the inner retina^18^. The degeneration of the inner retina could explain also the disruption of the retinal vasculature observed after 60 days from LD in the deep plexus. A longer time after LD (120 days), a recovery in the retinal vessel network, in terms of structure, was observed. However, a higher number of tufts was still present and the choroid adhered with the inner retina due to massive photoreceptor degeneration. VEGFA, bFGF and their receptors were up-regulated after 120 days and the amount was even higher than in earlier times of recovery. It is possible that the increase of pro-angiogenic factors is an attempt to restore the physiological vessel network of the neuroretina.

In retinal neoangiogenesis, a pivotal role is also played by the microglia^13^. In fact, although the interaction between microglia and blood vessels is important for a healthy retinal environment, when pathophysiological conditions occur, this interface contributes to the progression of the diseases and it culminates in the breakdown of blood vessels and to the sprouting of new branches^47^. Under stress conditions, microglia up-regulate the expression of VEGF^13,48^. We therefore hypothesized that the neovascularization phenomena were also related to microglia activation, which it is known to migrate to the photoreceptor layer and to the subretinal space in the light damaged model^19^. We demonstrated that activated microglia cells are still present after a long time from a brief light exposure, consistently with the progressive degeneration events observed in the retina. Moreover, activated microglia were found surrounding the new vessels, supporting the hypothesis that they are involved in angiogenesis alterations in our experimental model. Nevertheless, further studies need to be performed in order to deepen the knowledge on the role of microglia in the progression of retinal neovascularization, as well as the origin of the vessels infiltrating the photoreceptor layer. This work also adds an important interpretation of the retinal functional response observed after light damage. The electrophysiological result in fact has shown that already after 7 days of recovery from light damage the functional response decreases significantly compared to the healthy animals and it does not impair over time. In fact, there is not a direct relationship between photoreceptor death and the impairment of ERG responses. An explanation of this has already been described in other works in reference to the upregulation of inflammatory and self-protection factors^24,27,28^. Here, we demonstrated that the electrophysiological impairment of the retina in the LD model is also due to the neoangiogenic phenomena in the ONL and OPL and to the up-regulation of pro-angiogenic factors.

In conclusion, in this work we added relevant findings into the understanding of the degeneration processes following the exposure to acute light damage. We demonstrated for the first time that an albino Sprague Dawley rat exposed for 24 hours at 1000 lux undergoes retinal damage and develops neovascularization together with the up-regulation of relevant pro-angiogenic pathways and long-term microglia activation. Neovascularization in the photoreceptor layer, as well as the modulation of the pro-angiogenic signalings, occurs already a week after the induction of damage. After few months, retinal atrophy is reproduced as well and the choroid gets in touch with the neuroretina. The deepening of the new vessels origin and the investigation of the possible anastomosis between the choroid and the retinal vasculature with further future experiments will allow us to define whether the acute LD model can be used specifically as a model for neovascular AMD.

## Methods

### Animals

All animal experiments were conducted according to the ARVO statement for the use of animals in ophthalmic and vision research. All experiments were authorized by the Italian Ministry of Health, number 448/2016-PR of April 27, 2016.

Sprague Dawley (SD) albino rats were born and raised in dim cyclic light condition (12 hours light, 12 hours dark) with an ambient light level of approximately 5 lux. The animals had free access to food and water.

### Light damage

Animals selected for the experiments were placed in individual Plexiglas cages with food placed on the floor and water in plastic bottles. They were dark adapted overnight and exposed to 1000 lux at 9 a.m. for 24 h (LD24h). Thereafter, they were returned to dim cyclic light conditions for 7, 60 and 120 days respectively to recover from the acute stress due to light exposure. The LD24h group instead was sacrificed immediately after light exposure^18^.

On this basis the study was performed on 5 different groups:

1. Control (CTRL);
2. LD24h (LD);
3. LD24h + 7 days of recovery (LD + 7rec);
4. LD24h + 60 days of recovery (LD + 60rec);
5. LD24h + 120 days of recovery (LD + 120rec).

### Electroretinogram (ERG) recordings

In order to minimize variability among the five experimental groups, electrophysiological recordings (flash electroretinogram, fERG) were performed before exposing animals to high intensity light. This functional evaluation was necessary to select animals with similar starting retinal electrophysiological responses. The fERG recordings were performed at 7, 60 and 120 days after light exposure respectively. The fERG was recorded in a dark-adapted condition in response to a single white light flash of increasing intensity (scotopic), delivered by a standard Ganzfeld Stimulator (BiomedicaMangoni, Pisa Italy). Before performing fERG, the rats were anaesthetized with an intraperitoneal injection of Ketamine/Xylazine (10 mg/ 100 g–1.2 mg/100 g), mounted on a stereotaxic apparatus and the body temperature was maintained at 37.5 °C. Corneas were anaesthetized with a drop of novocaine and pupils were dilated with visumidriatic 1.0% tropicamide. The recordings were carried out for both eyes simultaneously with a gold electrode loop (2.0 mm in diameter) placed on the corneas. The reference electrodes were inserted subcutaneously in the proximity of the eyes and the ground electrode was inserted in the anterior scalp, between the eyes. The responses were recorded at increasing light intensities (0.001–100 cd*s/m^2^ range). At the end of each recording session, traces were bandpass filtered between 0.3 and 500 Hz. A- and b-waves amplitude (μV) for each luminance were measured. We also performed Oscillatory potentials (OPs) analysis, by calculating the sum of the amplitude of OP1, OP2, OP3 and OP4^18^.

### Cryosections

The rats were euthanized and the eyes enucleated for morphological analyses. The eyes were enucleated, fixed in 4% paraformaldehyde for 6 h and washed in 0.1 M phosphate buffered saline (PBS, pH 7.4). The cornea and lens were removed, the eyes were cryoprotected by immersion in 10%, 20% and 30% sucrose overnight, and finally they were embedded in the Tissue Tek OCT (optimum cutting temperature, Qiagen, Valencia, CA) compound for proper freezing in liquid nitrogen. Cryosections of 20 μm thickness were made for each retina and collected in gelatine and poly-l-lysine-coated slides. The sections crossing the optic nerve were chosen for subsequent analysis for all the experimental groups. All sections were stained with Bisbenzimide, to make visible the cell nuclei^18^.

### Immunofluorescence staining

To block non-specific binding sites, 5% bovine serum albumine (BSA), 10% Goat Serum (GS) or 0.75% Horse Serum (HS) were used. Sections were incubated overnight at 4 °C with primary antibodies: polyclonal anti-VEGFA (Santa Cruz, sc-7269) (1:250 in 1% BSA), polyclonal anti-VEGFR2 (Invitrogen, AHR5102) (1:250 in 1% BSA), polyclonal anti IBA-1 (Wako Pure Chemical Industries, 019-19741) (1:200 in 1% GS), monoclonal bFGF (Millipore 2718303) (1:200 in 0,75% HS), monoclonal FGFR1 (OriGene TA324059) (1:250 in 1% GS) and plyclonal anti-vWF (Chemicon, AB7356) (1:250 in 1% BSA). All the antibodies used in this paper are summarized in Supplementary Table S1. Secondary antibodies were anti-mouse or anti-rabbit IgG conjugated to red or green fluorescent dies (Alexa Fluor 594 or 488; Molecular Probes, Invitrogen, Carlsbad, CA) diluted 1:1000 and incubated at 37 °C for 2 h. Anti-mouse IgG conjugated to red fluorescent dye was used for anti-VEGFA; anti-mouse IgG conjugated to green fluorescent dye was used for anti-VEGFR2 and anti-bFGF; anti-rabbit IgG conjugated to red fluorescent dye was used for anti-FGFR1, anti-IBA-1 and anti-vWF. Images of immunolabeled cryosections were acquired by using a Leica TCS SP5 confocal microscope.

### Isolectin staining

In order to detect the vessels, Isolectin B4 staining was performed on retinal cryosections. To block non-specific binding sites, 10% goat serum (GS) was used. Sections were incubated overnight at 4 °C with Isolectin B4 (IB4) Alexa Fluor dye conjugates (1:200 in 1% GS). Since it is known that IB4 marks also activated microglia^49^, IB4 was also used together with anti-IBA1 immunostaining in order to detect any activation of the microglia cells, and together with anti-vWF to specifically detect the signal deriving from endothelial cells. Images were then acquired by using a Leica TCS SP5 confocal microscope.

### Retinal whole mounts

For whole mount analysis retinas were isolated from the eye, fixed in 4% paraformaldehyde, and washed with 0,1 M TrisHCl (pH 7.4). To block non-specific binding sites, 10% goat serum (GS) was used. Whole mounts were then incubated with Isolectin B4 Alexa Flour dye conjugates (1:150 in 1% GS) for 36 hours at 4 °C. The whole mounts were also labelled with nuclear staining Bisbenzimide in order to identify the retinal layers. They were then collected on gelatine and poly-l-lysine-coated slides. Images of Isolectin were acquired by using a Nikon Eclipse 80i confocal microscope. Vessels analysis was performed by the AngioTool software in order to quantify the amount of vessels in terms of “vessels percentage area”. The number of tufts of neovascularization was also quantified by counting them on the acquired images. Tufts were identified looking at a well defined swelling morphology both at the terminals and in the middle of the vessels. The analysis was performed on the vessels of the following plexuses: the superficial plexus (corresponding to GCL layer), the inner plexus (corresponding to the IPL and INL) and the deep plexus (corresponding to the OPL). The three different plexuses were discriminated by looking at the nuclei of the nervous cells. The vessels of each retinal layer were acquired at three different fields of the dorsal retina of each animal and the mean was calculated and compared between all groups.

### Western Blot

Total proteins were extracted from eye cup samples by using a Dounce Homogenizer and a lysis buffer (50 mM Tris-HCl pH 7.5, 1% Triton X-100, 0.1% SDS, EDTA 5Mm, Halt Protease and Phosphatase Inhibitor Cocktail, Thermo Fisher Scientific and QS dH2O). Bradford Assay (Bio-Rad Laboratories, Milan, Italy) was performed in order to quantify the protein content. 70 µg of the protein extracts were run on a Bolt 4-12% Bis-Tris Plus (Thermo Fisher Scientific) at 200 V for 20 minutes. The proteins were transferred to PVDF membrane (Millipore, Milan, Italy) through a iBlot 2 Dry Blotting System (Invitrogen IB21001).

After blocking at RT for 1 h with 5% nonfat dry milk in TBST, the membranes were washed briefly and incubated with primary antibodies directed either against VEGF (Santa Cruz sc-7269), VEGFR2 (Invitrogen, AHR5102), bFGF (Merck, 05-117), FGFR1 (OriGene, TA324059) and Tubulin (Thermo Scientific, 62204) diluted in 5% non fat dry milk in TBST. All the antibodies used in this paper are summarized in Table S1 reported in supplementary informations. Afterward, the membranes were incubated for 1 h at RT with the specific HRP-conjugated secondary antibody (anti-rabbit or anti-mouse) and then incubated in SuperSignal West Pico Plus (Thermo Fisher Scientific Inc) chemiluminescent substrate and detected using a ChemiDoc XRSplus imaging system (Bio-Rad Laboratories). The optical densities of blot bands were obtained by ImageJ (U.S. National Institutes of Health, Bethesda, Maryland, USA) software analysis and normalized versus tubulin as internal control^50^.

### Statistical analysis

Statistical analysis was performed by one-way ANOVA test followed by Tukey test. First type error was set at 5%. The statistical analysis was conducted using the SigmaPlot 12.0 software. Data are shown as mean ± SE.

## Supplementary information

Supplementary information.
