# Supplementary material for: Up-regulation of pro-angiogenic pathways and induction of neovascularization by an acute retinal light damage
Source: Sci Rep. 2020 Apr 14;10:6376. doi: 10.1038/s41598-020-63449-y (PMC7156521; doi:10.1038/s41598-020-63449-y)
Supplement: Supplementary file 1 — Supplementary information. [file 41598_2020_63449_MOESM1_ESM.pdf]

Up-regulation of pro-angiogenic pathways and induction of neovascularization by an acute retinal light damage

Annamaria Tisi*, Giulia Parete*, Vincenzo Flati, and Rita Maccarone
*Joint first authorship

SUPPLEMENTARY FIGURES

Supplementary Figure S1

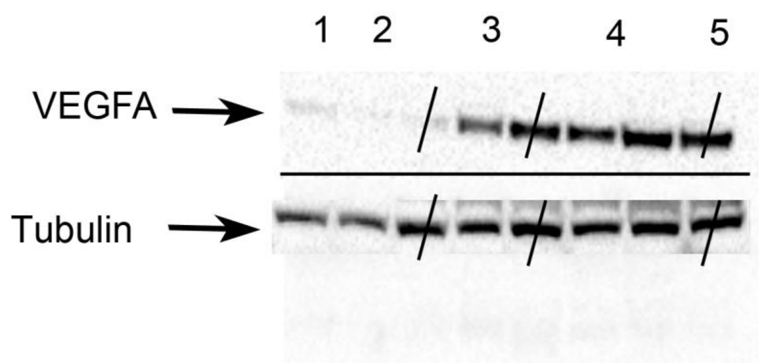

Supplementary Figure S1: Original whole western blot shown. For the final figure, photoshop was used to flip the western blot horizontally and crop the horizontal line of VEGFA/Tubulin bands indicated by the arrows. They were then placed in the Figure 2A.

Supplementary Figure S2:

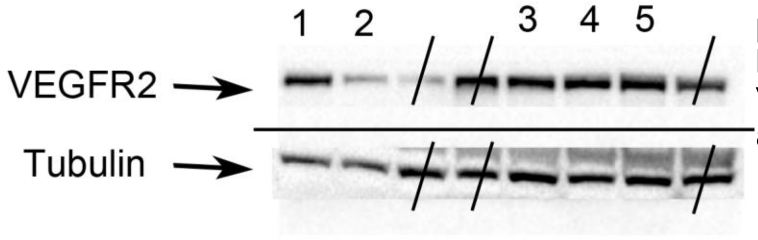

Supplementary Figure S2: Original whole western blot shown. For the final figure, photoshop was used to flip the western blot horizontally and crop the horizontal line of VEGFR2/Tubulin bands indicated by the arrows. They were then placed in the Figure 3A.

Supplementary Figure S3:

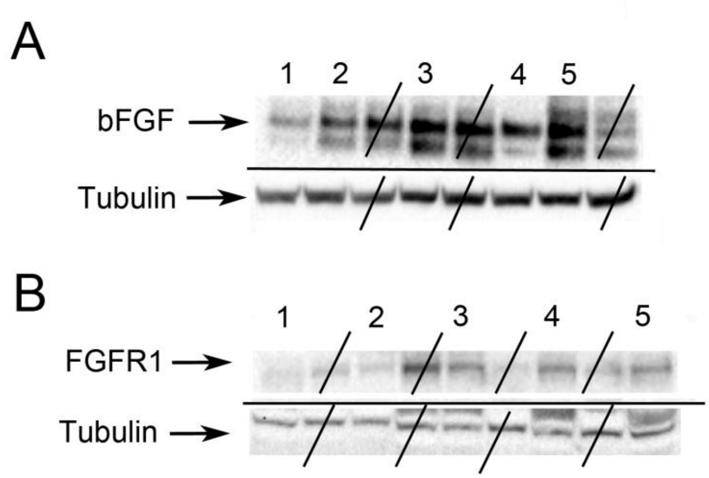

Supplementary Figure S3 A: Original whole western blot shown. For the final figure, photoshop was used to flip the western blot horizontally and crop the horizontal line of bFGF/Tubulin bands indicated by the arrows. They were then placed in the Figure 4A.

Supplementary Figure S3 B: Original whole western blot shown. For the final figure, photoshop was used to flip the western blot horizontally and crop the horizontal line of FGFR1/Tubulin bands indicated by the arrows. They were then placed in the Figure 4A.

Supplementary Figure S4:

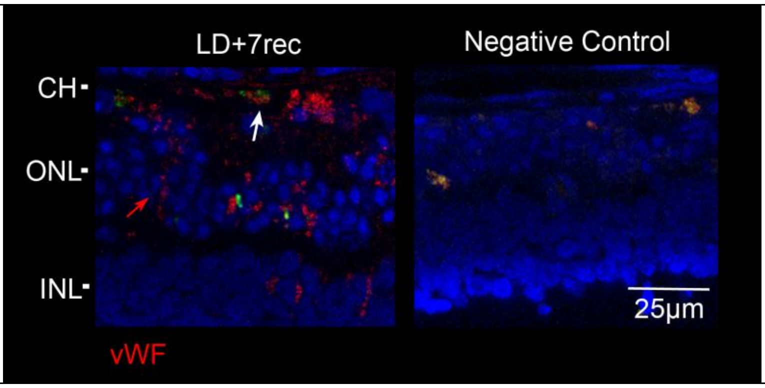

Supplementary Figure S4: Left: representative confocal image of anti-vWF immunostaining (red) 7 days after light damage, acquired with both 594 and 488 excitation wavelengths to discriminate vessels (red arrow) from autofluorescent debris (white arrow). Right: Negative control without primary antibody.

Supplementary Figure S5:

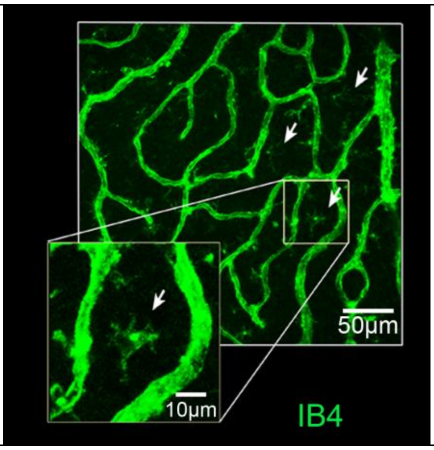

Supplementary Figure S5: microglia surrounding the retinal vessels. Representative image of a whole mount retina from LD+7rec group stained with Isolectin B4 (IB4) (green). The white arrow indicates activated microglia binding IB4.

Supplementary Table S1: Summary of the antibodies used in this paper

Antibody	Species	Company, Catalog#	Use
VEGFA	mouse	Santa Cruz, sc-7269	IF, WB
VEGFR2	mouse	Invitrogen, AHR5102	IF, WB
bFGF	mouse	Millipore, 2718303	IF
bFGF	mouse	Merck, 05-117	WB
FGFR1	rabbit	OriGene TA324059	IF, WB
IBA-1	rabbit	Wako Pure Chemical industries, 019-19741	IF
Tubulin	mouse	Thermo scientific, 62204	WB
vWF	rabbit	Chemicon, AB7356	IF
